# Ventricular fibrillation during football training as a consequence of kratom and caffeine use in an adolescent: case report

**DOI:** 10.1093/ehjcr/ytae364

**Published:** 2024-07-24

**Authors:** Jozef Dodulík, Jiří Plášek, Petr Handlos, Andrea Gřegořová, Jan Václavík

**Affiliations:** Department of Internal Medicine and Cardiology, University Hospital Ostrava, Ostrava, Czech Republic; Department of Internal Medicine and Cardiology, University Hospital Ostrava, Ostrava, Czech Republic; Centre for Research on Internal and Cardiovascular Diseases, Faculty of Medicine, University of Ostrava, Ostrava, Czech Republic; Institute of Forensic Medicine, University Hospital Ostrava, Ostrava, Czech Republic; Faculty of Medicine, Institute of Forensic Medicine, University of Ostrava, Ostrava, Czech Republic; Department of Medical Genetics, University Hospital Ostrava, Ostrava, Czech Republic; Department of Internal Medicine and Cardiology, University Hospital Ostrava, Ostrava, Czech Republic; Centre for Research on Internal and Cardiovascular Diseases, Faculty of Medicine, University of Ostrava, Ostrava, Czech Republic

**Keywords:** Kratom, Mitragyna speciosa, Mitragynine, Ventricular fibrillation, Implantable cardioverter-defibrillator, MYOM1, Case report

## Abstract

**Background:**

There is an increase in the sale of legal drugs in our country. One of these substances is kratom. Kratom (*Mitragyna speciosa*) is a partial agonist of the opioid kappa, mu, and delta receptors. It acts as a stimulant at low concentrations, making users feel more energetic and euphoric. It has sedative and antinociceptive effects at higher doses.

**Case summary:**

An 18-year-old man collapsed during football training and required cardiopulmonary resuscitation; the initial rhythm was ventricular fibrillation managed by defibrillation. Laboratory parameters were unremarkable. Blood samples sent for toxicological evaluation were positive for kratom and caffeine. Echocardiographic examination, coronary computed tomography angiography, and cardiac magnetic resonance imaging did not prove the cause. Genetic testing did not find a pathogenic gene variant associated with familial ventricular fibrillation, but a variant of unknown significance was found in MYOM1. Given this situation, we implanted an implantable cardioverter-defibrillator (ICD) from the secondary prevention of sudden cardiac death (SCD) according to the guidelines of the European Society of Cardiology (ESC). No recurrence of ventricular arrhythmia has been reported by ambulatory ICD memory checks on our patient.

**Discussion:**

In some country, kratom is freely available and sold as a plant, not a drug. Only incident cases of ventricular fibrillation after kratom use are described in the literature. There is insufficient scientific evidence linking kratom to ventricular fibrillation. This is an absolutely crucial case report of this type, which has not yet been published in similar circumstances in the world. Therefore, the development of ventricular fibrillation was assumed to be due to a combination of kratom, caffeine, and exercise. The safety profile and effects of kratom should be the subject of future research. We would like to stress the importance of reporting further case series for more scientific evidence and thus increasing the pressure for stricter availability and regulation of kratom in some countries, especially where it is over-the-counter.

**Learning points**

- Kratom has potentially cardiotoxic arrhythmogenic effects.
- Kratom use can cause dependence and withdrawal symptoms upon discontinuation.
- Kratom should not be combined with other stimulants.
- Unless proven as secondary to a reversible cause, idiopathic ventricular fibrillation is an indication for implantation of a cardioverter-defibrillator for secondary prevention of sudden cardiac death.

## Introduction

The first records of kratom (*Mitragyna speciosa*) date back to the 19th century in the region of Indochina, where the plant is found. Forty chemical substances have been isolated from kratom leaves. However, only four alkaloids are pharmacologically active: mitragynine, 7-hydroxy mitragynine, corynantheidine, and speciociliatinine.^1^

Kratom is thought to act on the opioid kappa, mu, and delta receptors as a partial agonist.^2^ However, the mechanism is not precisely understood. At low concentrations, it is a stimulant, making users feel more energetic to euphoric. At higher doses, it is a sedative, antinociceptive, spasmolytic, and antidiuretic. It may also cause dysphoria, depression, and insomnia. The effects after ingestion take 5–10 min and persist for 2–5 h. The use of kratom can cause dependence and withdrawal symptoms upon discontinuation. An *in vitro* study on a pluripotent stem cell colony, Lu *et al*.^3^ demonstrated the inhibition of specific hERG1a/1b potassium channels by mitragynine. These channels are important in the regulation of cardiac rhythm. If these channels are blocked, the QT interval may be prolonged, which is one of the elementary indicators of torsade de pointes.

The toxic concentration of kratom in plasma is unknown. In experiments on laboratory animals, lethal concentrations of mitragynine have been determined above 230 ng/mL.^4^ Kratom should not be combined with other stimulants, such as cocaine, amphetamine, or caffeine, for further potentiation of its effects.

## Summary figure

**Figure:**
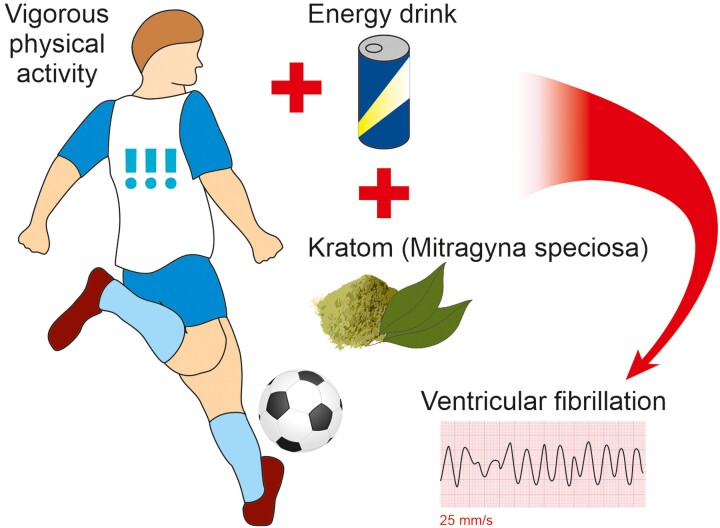


## Case presentation

**Figure 1:**
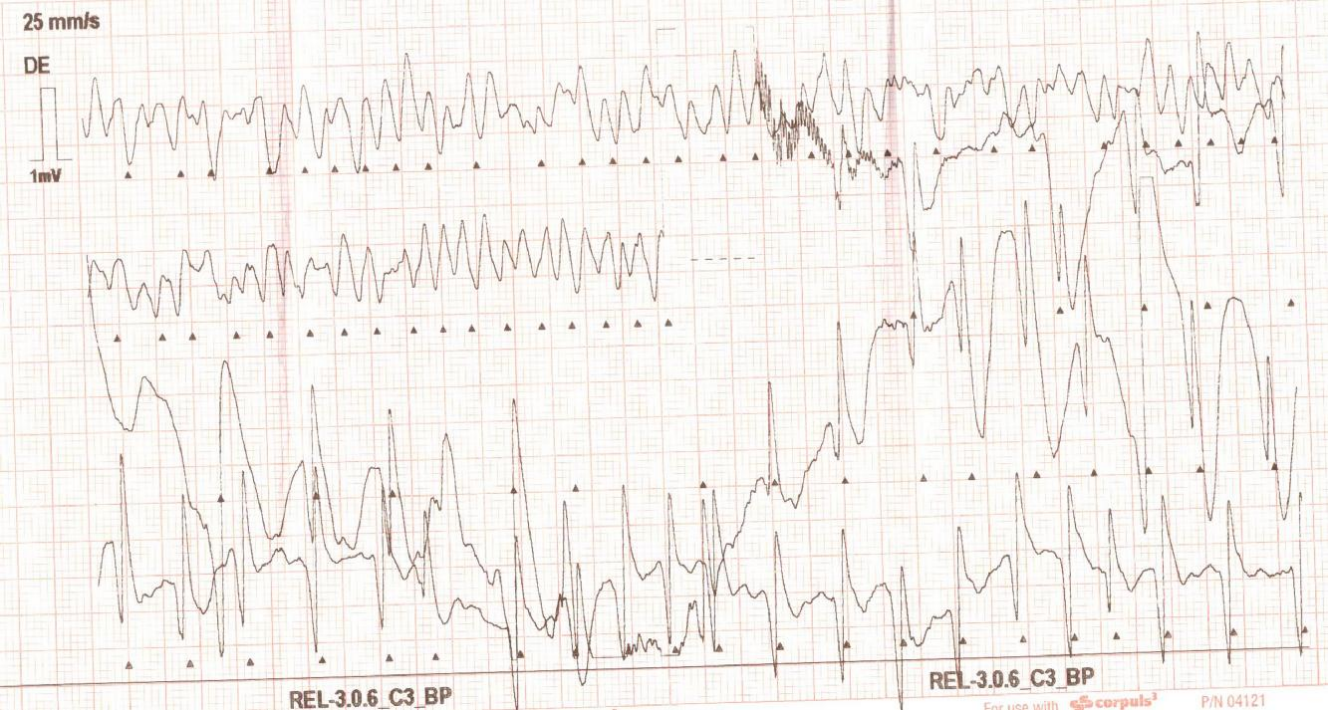
Electrocardiogram pattern of ventricular fibrillation upon arrival of the emergency medical service.

An 18-year-old male collapsed during football practice; according to witnesses present, there were full-body convulsions, breathlessness, and gasping. Promptly, the ambulance crew initiated professional cardiopulmonary resuscitation. The initial rhythm was ventricular fibrillation (*Figure 1*), and defibrillation was performed with a single 200 J biphasic shock, after which sinus rhythm, circulation, and consciousness were restored. The patient was agitated, and recurrent ventricular tachycardia with a heart rate of 180 b.p.m. was detected on the telemeter, blood pressure was 110/60 mmHg, oxygen saturation was 92%, and respiratory rate was 20 b.p.m. The patient was intubated and transferred to the hospital for further management. After transfer to the hospital, the patient was extubated early (after 24 h). There was no nosocomial infection or other compilation, and the patient was free of neurological deficit after regaining consciousness.

The patient was Caucasian, an active athlete, previously untreated, not taking medication, and had no family history of cardiovascular disease. Initial laboratory parameters were unremarkable except for elevation of high-sensitivity troponin I (hs-TnI) 230 ng/L (0–53.5 ng/L). Due to the patient’s age, blood samples were sent for qualitative toxicology evaluation.

**Figure 2:**
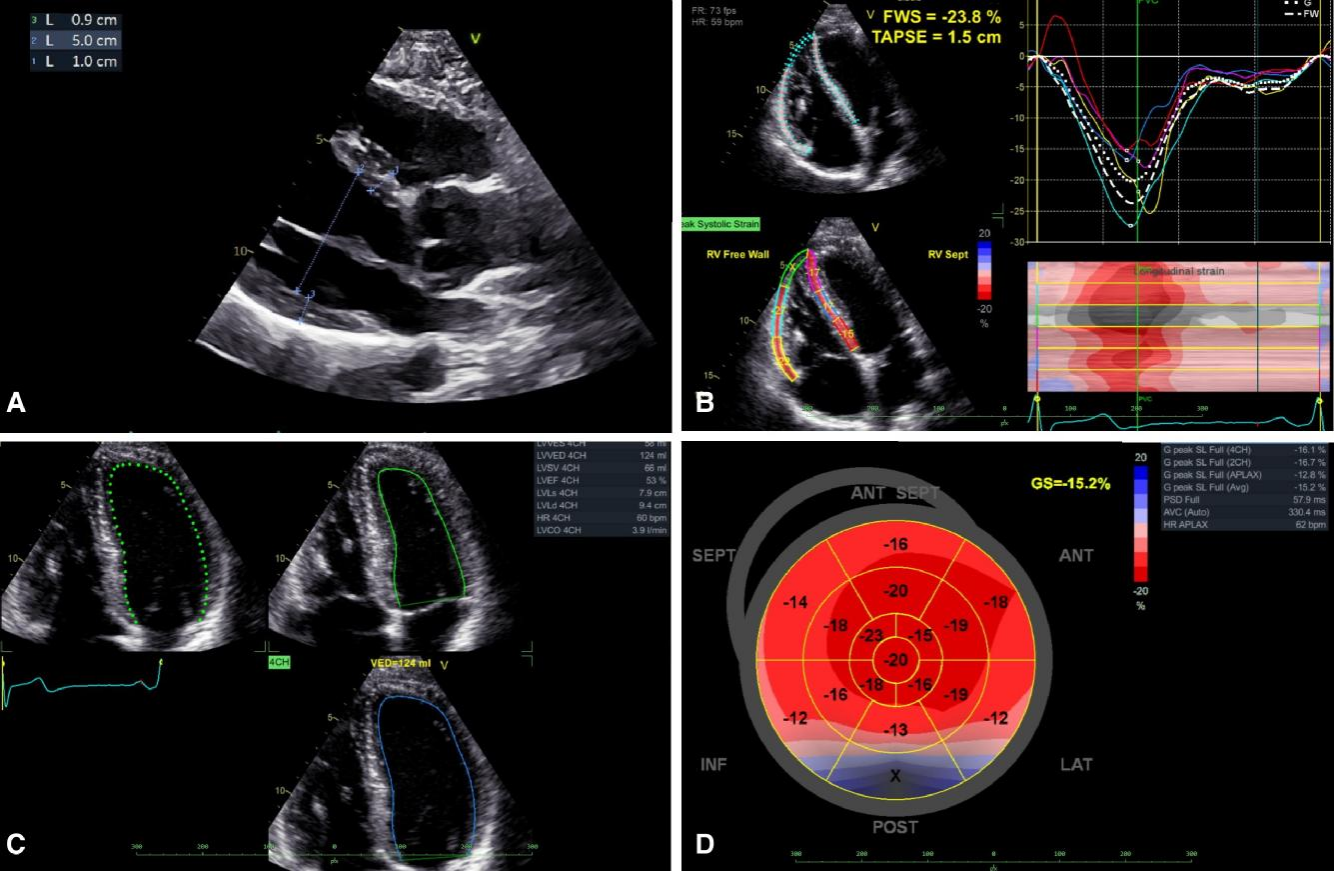
Echocardiographic examination: (*A*) long axis with normal left-sided heart compartments and aorta. (*B*) Normal non-dilated right ventricular function. (*C*) Normal left ventricular function. (*D*) No regional kinetics disturbance.

**Figure 3:**
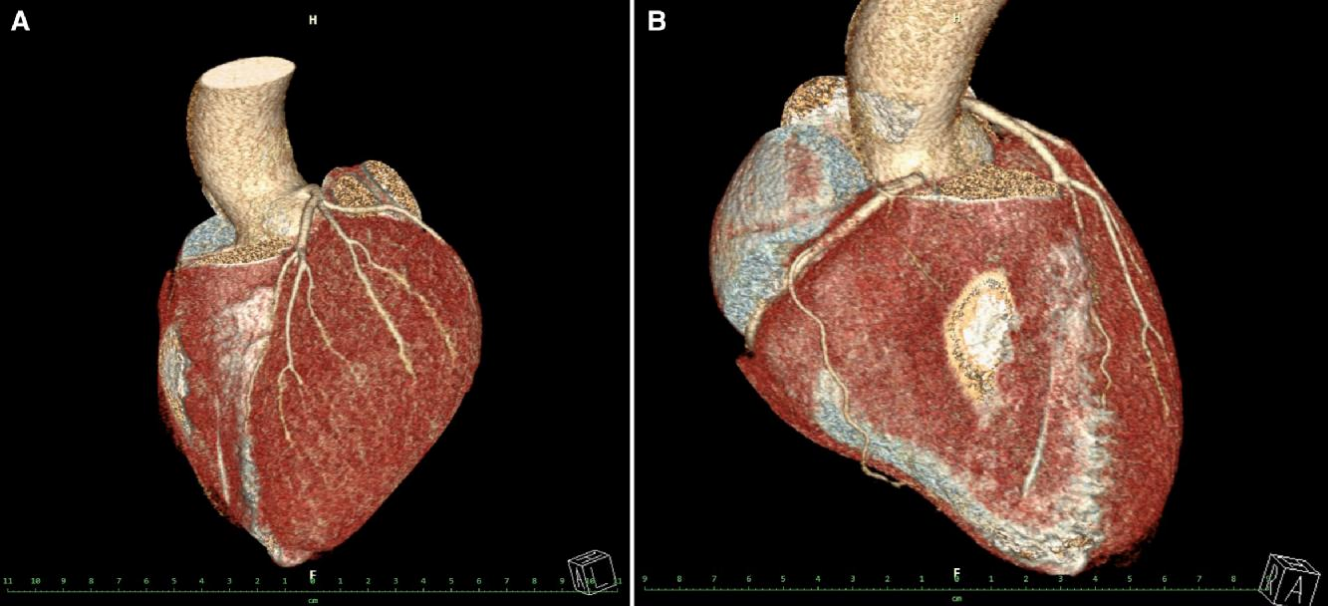
3D reconstruction of coronary computed tomography angiography. (*A*) Displaying left main coronary vessel and its branching (arrow) with no obstruction or other pathology. The dimensions and structure of other heart chambers are within range. Also aortic root is not dilated. (*B*) Showing the right coronary artery territory without obvious pathology.

The toxicology examination was positive for plasma kratom and caffeine, and the mitragynine level was 98 ng/mL. It was subsequently confirmed by the patient that, on the day of the incident, he had consumed 250 mL of an energy drink to which he had added 2 g of kratom in powder form before football practice. Echocardiographic examination showed good left and right ventricular function, with no dilatation of the right-sided ventricles and no significant valvulopathy (*Figure 2*). An ischaemic aetiology of the ventricular arrhythmias seemed unlikely because of the patient’s young age; a coronary artery anomaly may be a slightly more likely aetiology. However, coronary computed tomography angiography showed neither coronary heart disease nor coronary artery anomaly (*Figure 3*).

Cardiac magnetic resonance imaging was also performed, which did not detect structural cardiac involvement. There was no recurrence of ventricular arrhythmia during telemetry monitoring. As part of the differential diagnosis, an ajmaline test was performed to exclude Brugada syndrome.

Furthermore, genetic testing of the patient’s genomic DNA based on the classical Sanger DNA sequencing method was performed by a panel of 263 genes with a focus on heart disease. No pathogenic or likely pathogenic sequence variants in any of the genes associated with familial ventricular fibrillation were found. Copy number variation analysis did not identify the cause of the disease at the molecular level. A next-generation sequencing panel evaluation identified sequence variant c.3944C>G(p.Thr1315Arg) in MYOM1 in a heterozygous state, which is listed as a variant of unknown significance in the NCBI ClinVar database.

**Figure 4:**
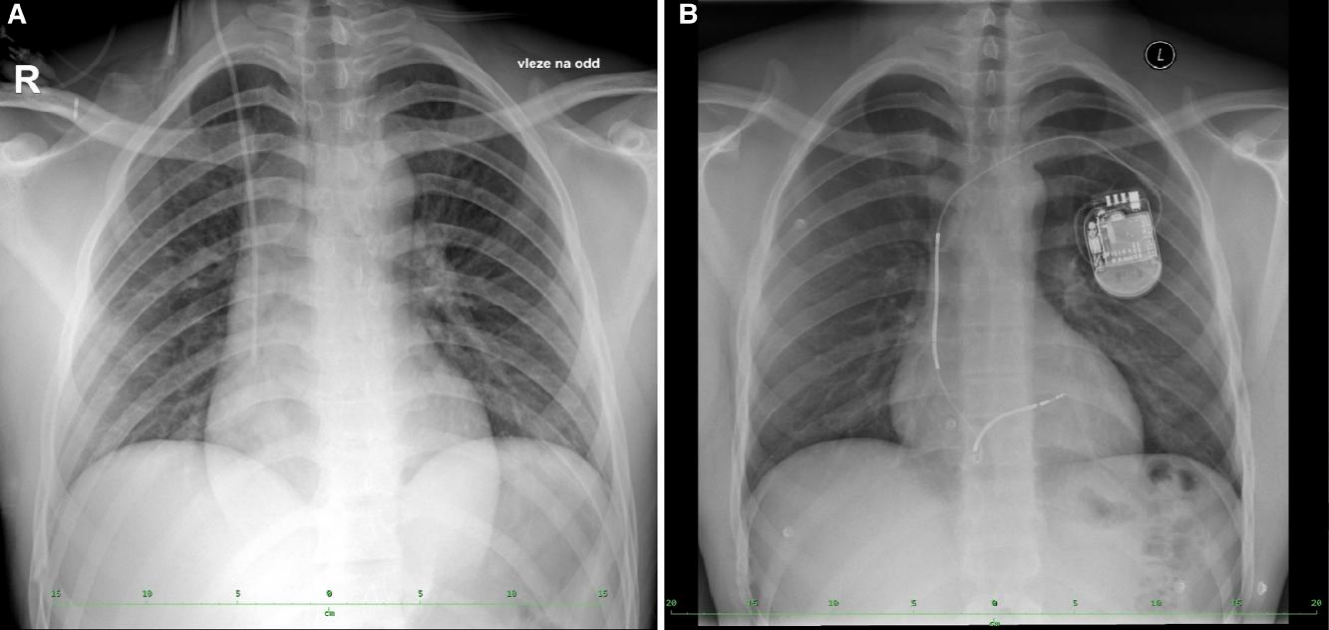
Chest X-ray: (*A*) Upon arrival at the hospital, supine image. (*B*) After implantation of the implantable cardioverter-defibrillator.

According to the ESC guidelines, ICD is indicated in patients with documented ventricular tachycardia after excluding reversible causes in Class I Level A recommendations.^5^ This patient was indicated for implantation of a one-cavity ICD for secondary prevention of SCD (*Figure 4*). The procedure was performed normally, without complications, and the patient was readmitted to outpatient care after an 11-day hospital stay. The patient and his family were thoroughly educated about the dangers of kratom and energy drink use. Absolutely no exposure to these products was recommended.

The patient was transferred to the arrhythmology outpatient clinic and is no longer taking kratom. No recurrence of ventricular arrhythmia was detected in the ICD memory at the outpatient follow-ups at 1, 3, and 6 months after diming.

## Discussion

In the age group over 35 years, coronary atherosclerosis is clearly the leading cause of SCD in athletes, whereas genetic causes or coronary anomalies dominate in younger athletes. Of the gene-related causes of athlete SCD, hypertrophic cardiomyopathy dominates, with arrhythmogenic cardiomyopathy next in order. Other important gene-related causes of SCD are long QT interval syndrome and catecholaminergic polymorphic ventricular tachycardia. Also myocarditis must be included among the possible causes of SCD in athletes. Rarer aetiologies include aortic aneurysm rupture, aortic valve stenosis, short QT interval syndrome, Brugada syndrome, or arrhythmogenic right ventricular cardiomyopathy.^6^ These diseases were excluded in our patient.

Kratom is over-the-counter in Czech Republic, unlimited by age and quantity. It is sold in the form of raw leaves, tea, tablets, or powder. Sellers’ state on their labels that it is a substance sold ‘for collectors’ purposes only’. It can be purchased in vending machines, in brick-and-mortar stores, or in e-shops. The price of kratom in Czech Republic currently ranges from 4 to 6 EUR per 25 g pack depending on the type and quality of the product. The level of experience with kratom has been increasing in recent years, especially among adolescents. According to a questionnaire survey among university students in Czech Republic, one-fifth to one-third of students have experience with kratom, and its popularity continues to grow.^7^

Only incident cases of ventricular fibrillation or polymorphic ventricular tachycardia after kratom use have been described in the literature.^8,9^ There is insufficient scientific evidence linking kratom alone to ventricular fibrillation. A review by Leong *et al*.^10^ on the cardiotoxicity of kratom suggested that the most common adverse effects are hypertension (11%) and tachycardia (25%). Although an *in vitro* study has demonstrated a risk of QT interval prolongation and polymorphic ventricular tachycardia, such as torsade de pointes, after kratom use, this effect has not been clearly demonstrated in people taking kratom regularly.^3,10^

In the absence of genetic testing results, which are usually only available after a few weeks, we were faced with the choice of how to proceed in this case. From the outset, it was not clear whether the development of ventricular fibrillation was due to kratom, the effects of which could have been accentuated by the use of a caffeinated energy drink and physical exertion during football training, or to a genetic mutation that had not yet been identified. However, according to the ESC guidelines, if ventricular fibrillation is detected, implantation of an ICD is indicated as soon as possible.^5^

Within 8 weeks, the results of genetic testing were delivered and showed no known pathogenic mutation. A sequence variant, c.3944C>G(p.Thr1315Arg), was found in MYOM1 in a heterozygous state, which was evaluated as a variant of unknown significance. This variant is currently not described in the literature. The clinical significance of the sequence changes found in MYOM1 is currently under investigation, especially in patients with hypertrophic or dilated cardiomyopathy or in patients with sudden cardiac death. Currently, the degree of pathogenicity of this sequence variant cannot be determined.

The patient was transferred to the arrhythmology outpatient clinic and is no longer taking kratom. No recurrence of ventricular arrhythmia was observed by regular checks of the ICD memory. Therefore, it is reasonable to assume that, in this particular case, the ventricular fibrillation developed as a result of a combination of kratom, a caffeinated energy drink, and physical exertion.

We do not know to what extent kratom alone, or its combination with other stimulants, can be evaluated as an unequivocal reversible aetiology of ventricular fibrillation. Until the exact mechanism of action on the cardiovascular system is elucidated and the toxic dose of kratom or mitragynine causally associated with arrhythmogenic risk is determined, it will be necessary to follow guidelines for the implantation of an ICD from secondary prevention in idiopathic ventricular fibrillation, as in our case.^10^

The main educational insights of this case can be summarized as follows:

1. Kratom itself or in combination with other stimulants is potentially dangerous during strenuous exercise.
2. Kratom sales should be regulated on the national or European level to avoid its use by kids and young adults.
3. Other cases or case series need to be reported to validate our observation.
4. Unless a direct link between kratom use and ventricular fibrillation will be established, patients need to be implanted with an ICD in the secondary prevention of SCD according to the ESC guidelines.^5^

## Conclusion

This communication’s main aim is to warn about the potentially dangerous combination of kratom with other energy products, which can lead to serious cardiovascular complications with potentially fatal outcomes. We think that our case could contribute to the process of delegitimization of kratom in our country and its inclusion in the list of banned addictive substances.

## Data Availability

The data underlying this article are available in the article and in its online supplementary material.

## Lead author biography

I work as a doctor at the cardiovascular clinic at the University Hospital in Ostrava, where I mainly deal with patients with heart failure. I also work in the internal intensive care unit at Karvina Hospital and in a private cardiology and angiology ambulance in Ludgerovice. At the moment, I am preparing for cardiology certification. In the future, I would like to focus on arrhythmology.

## Notes

**Consent:** The patient consents to the publication of the case report in accordance with the COPE guidelines.

**Funding:** This case report was supported by the Ministry of Health CZ-DRO (FNOs/2023).
